# Wild bee diversity in the Entre-Sambre-et-Meuse National Park (Belgium)

**DOI:** 10.3897/BDJ.14.e176439

**Published:** 2026-01-26

**Authors:** Maxence Gérard, Vélinka Beaubois, William Fiordaliso, Félicien Gautier, Arielle Guillaume, Jeanne Peduzzi, Guillaume Ghisbain

**Affiliations:** 1 Laboratory of Zoology, Research Institute for Biosciences, University of Mons, Place du parc 20, 7000 Mons, Belgium Laboratory of Zoology, Research Institute for Biosciences, University of Mons, Place du parc 20 7000 Mons Belgium; 2 Laboratory of Interaction Ecology and Global Change, Research Institute for Biosciences, 7000 Mons, Belgium Laboratory of Interaction Ecology and Global Change, Research Institute for Biosciences 7000 Mons Belgium; 3 Entre-Sambre-et-Meuse National Park, Route de Dailly 1, 5660 Couvin, Belgium Entre-Sambre-et-Meuse National Park, Route de Dailly 1 5660 Couvin Belgium

**Keywords:** Calcareous grassland, inventory, national park, pollinator, Red List

## Abstract

Human-induced environmental changes are driving declines in wild bee populations globally, threatening both pollination services and overall ecosystem stability. The implementation of effective conservation strategies for these pollinators ultimately depends on a clear understanding of both their local patterns of diversity and habitat associations. The Entre-Sambre-et-Meuse National Park (ESEMNP), recently created in southern Belgium, is situated in a botanically rich area but remains poorly documented in terms of standardised bee surveys. To address this gap, we monitored 32 sites over a five-month period, collecting 1159 specimens from 102 species. Of these, 17 are listed as threatened in Belgium’s most recent Red List, including four Critically Endangered species. Analyses revealed that calcareous grasslands supported the highest overall diversity and the greatest concentration of threatened taxa. These results emphasise the conservation value of certain habitat types within the park and the need for management practices that sustain both species richness and populations of at-risk species.

## Introduction

**Figure 1.**
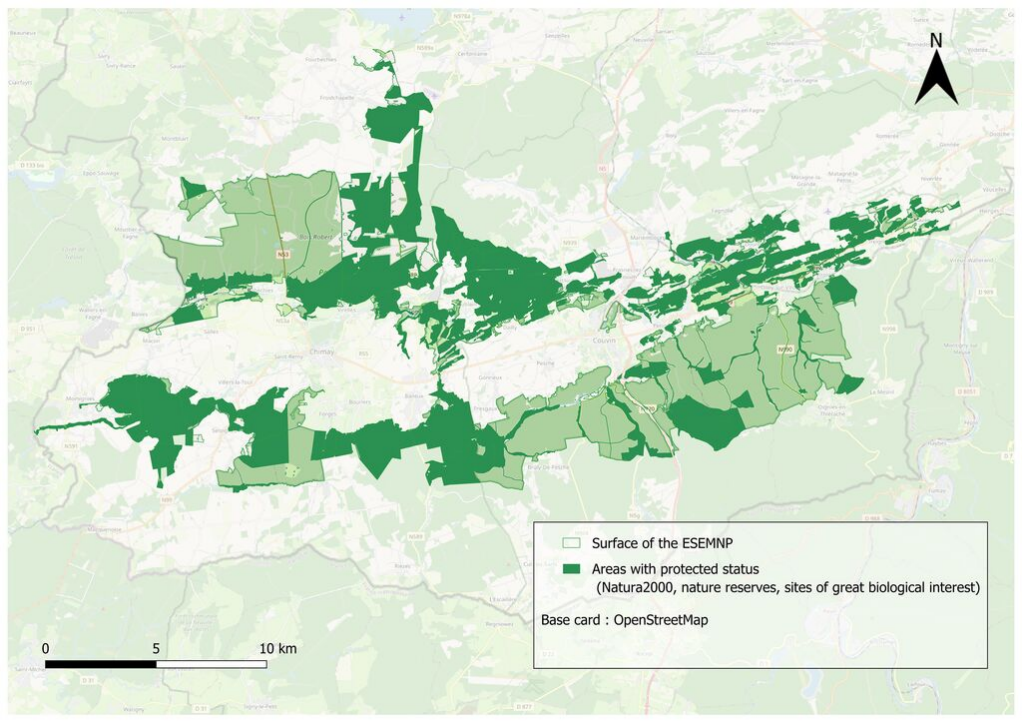
Distribution of sites of great biological interest (SGIB) and protected areas located in the Entre-Sambre-et-Meuse National Park (Belgium), including Natura 2000 sites and nature reserves.

With biodiversity declining at an unprecedented pace, conservation has become a global priority for governments and organisations seeking to safeguard ecosystems and secure a sustainable future (Diaz et al. 2019). In line with this objective, a call for projects in Wallonia, Belgium, led to the creation of the region’s first two National Parks. One of them, the Entre-Sambre-et-Meuse National Park (ESEMNP), was officially designated on 9 December 2022. Spanning 22,129 hectares across the provinces of Hainaut and Namur, the ESEMNP is renowned for its extensive forests, species-rich calcareous grasslands, and river systems such as the Viroin and the Eau Blanche. Its territory covers three distinct geological regions - the Ardenne, the Calestienne, and the Fagne - each contributing to the park’s unique ecological diversity. Habitats identified through the European Nature Information System (EUNIS) include perennial calcareous grasslands, sub-Atlantic lowland hay meadows, and tall-herb communities of humid habitats. Calcareous grasslands are particularly noteworthy in Wallonia due to their rarity and high conservation value (Adriaens et al. 2006). These habitats develop on calcium-rich soils and support a unique assemblage of plant and insect species, many of which are specialised and often threatened (WallisDeVries et al. 2002). The park also benefits from multiple conservation designations, spanning 7,038 hectares (approximately 31.8% of its area; Fig. 1) and encompassing 13 Natura 2000 sites.

Pollinators are responsible for ca. 78%-90% of the pollination of flowering plants worldwide (Ollerton et al. 2011, Tong et al. 2023), playing a pivotal role in maintaining wild plant communities and sustaining agricultural productivity (Potts et al. 2010). The economic importance of pollinators in Wallonia is also substantial: pollinator-dependent crops contributed an estimated €25.63 million in 2010, including €10.08 million in the provinces of Hainaut and Namur (Jacquemin et al. 2017). Bees are among the most important of these pollinators, yet their populations have undergone widespread declines in recent decades (Biesmeijer et al. 2006, Nieto et al. 2014, Rasmont et al. 2021, Zattara and Aizen 2021, Ghisbain et al. 2023a), driven by the combination of intensive pesticide use, land-use and management changes, climate change, interspecific competition with managed bees, and the spread of pests and pathogens (Potts et al. 2010, Goulson et al. 2015, Geslin et al. 2017, Gérard et al. 2020, Dicks et al. 2021, Ghisbain et al. 2025, Gekière et al. 2025). Belgium harbours 419 bee species, 389 of which have recent occurrence data (Vertommen et al. 2024), representing around 20% of the European diversity (Ghisbain et al. 2023b, Reverté et al. 2023). In certain regions, more than a century of field sampling has produced detailed inventories and valuable insights into population trends (Rasmont et al. 2005, Drossart et al. 2019, Vray et al. 2019, Rollin et al. 2020, Rasmont et al. 2021, Schatz et al. 2021). Yet, wild bee diversity has declined markedly over the past century. Among bumblebees - the best-studied group in Belgium - the number of species has decreased by 25.2% when comparing records from before and after 1950 (Rasmont et al. 2005, Rollin et al. 2020). Moreover, 45 bee species are currently listed as “Regionally Extinct” — though a few have since been rediscovered (Vertommen et al. 2024) — and 113 are classified as threatened according to the Belgian Red List (Drossart et al. 2019). 9.4% of the species are classified as “Data Deficient (DD),” a status assigned to species for which taxonomic uncertainty or limited and sporadic data prevent a reliable assessment of their extinction risk. This proportion is particularly low compared to the European average (56.7% DD, Nieto et al. 2014), suggesting that the Belgian bee fauna is relatively well studied. However, these broad figures do not imply a detailed knowledge of the different Belgian regions and species. Within this context, the Entre-Sambre-et-Meuse National Park (ESEMNP) emerges as a particularly valuable area. Based on citizen science records ranging from 1937 to 2025 (https://observations.be/), its habitats support 239 bee species, more than half of Belgium’s total diversity, making it a key region for both biodiversity conservation and ecological research.

Tackling the decline of wild bees requires targeted conservation initiatives, including the creation of National Parks and the implementation of standardised monitoring protocols (Drossart and Gérard 2020). In Wallonia, however, past scientific inventories have been limited and often lacked consistent methodology. A recent study provided a first standardized dataset for the Semois Valley National Park (SVNP) — the only other National Park in Wallonia — covering four habitat categories over five months of sampling (Gérard et al. 2025). While participatory citizen science platforms (https://observations.be/) provide valuable insights into the wild bee fauna of the Entre-Sambre-et-Meuse National Park (ESEMNP), these observations are not systematically verified (Turley et al. 2024). Similarly, although a few naturalist surveys have been conducted in select communes (Vandaudenard 2023), no scientific study has yet applied a standardised protocol linking wild bee species to specific habitat types. Within this context, the present preliminary monitoring aims to characterise these understudied bee populations and contribute to the long-term strategic and operational goals of the ESEMNP. By establishing a reliable baseline of local bee diversity and habitat associations, this work will provide essential guidance for future conservation and biodiversity management efforts within the park.

This paper presents the results of a standardised wild bee inventory carried out in spring and summer 2025 across diverse habitat types within the ESEMNP. We highlight the habitats that are particularly important for notable bee species, including those listed as threatened on the Belgian Red List or legally protected in Belgium, and provide insights into the specific habitat associations of these species.

## Material and Methods

**Selection of sampling sites**

**Figure 2.**
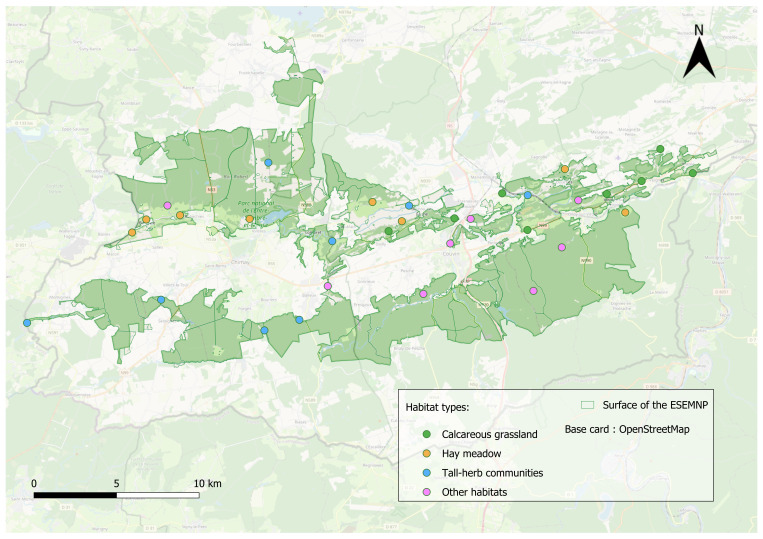
Distribution of the 32 sampling sites in the Entre-Sambre-et-Meuse National Park, Belgium. The colour code represents the habitat type at each sampling site.

Site selection aimed to capture the full range of habitats within the Entre-Sambre-et-Meuse National Park (ESEMNP) to provide a representative estimate of wild bee diversity. A total of 32 sites were selected based on their expected diversity of both plants and bees (Fig. 2). To reduce the risk of spatial autocorrelation, sampling locations were separated by a minimum distance of one kilometre in most of the cases, and 900m for two pairs of sites. This spacing reduces the likelihood that the same individuals would be present at more than one site, thereby preventing redundant sampling (Gathmann and Tscharntke 2002). Site areas varied between 0.18 ha and 10.82 ha (mean = 1.87 ha, median = 1.17 ha). The choice of sites was made in coordination with the ESEMNP management team, based on N2000 maps and EUNIS codes. Prior to fieldwork, owners of private plots were notified, and staff from the Département Nature et Forêt (DNF) were consulted regarding areas under public or state ownership. Authorisation for specimen collection was formally granted for each site by the Service Public de Wallonie (SPW).

Most of the selected habitats are classified using EUNIS codes, reflecting their ecological characteristics. Among the habitats particularly favourable to bees, perennial calcareous grasslands (EUNIS E1.2) are semi-natural thermophilous grasslands that develop on soils rich in calcium carbonate. These habitats are characterised by a high diversity of herbaceous species, often including many calcicolous plants. The vegetation is typically low-growing, dominated by grasses and flowering herbs, and maintained by traditional management practices such as grazing, which prevent encroachment by shrubs and trees. Sub-Atlantic lowland hay meadows (E2.22) are semi-natural grasslands typically found in lowland regions with a mild, humid climate. These meadows develop on fertile, well-drained soils and are traditionally managed through annual mowing for hay, often followed by light grazing. The vegetation is dominated by a mix of grasses and broad-leaved herbs, providing high structural and floral diversity. Perennial tall herbs lining watercourses (E5.41) are plant communities typically found in nutrient-rich, moist to wet soils. They are characterised by tall, dense stands of perennial herbaceous vegetation that develop along riverbanks, stream margins, ditches, and other wet linear habitats. These communities usually develop in areas where traditional agricultural management has been abandoned. These first three categories represent the main EUNIS habitat types within the National Park. Several additional habitat categories complement this list. Permanent mesotrophic pastures (E2.1) which are maintained under regular grazing, may provide additional resources for bees and contribute to habitat diversity. Other notable habitats include *Salix* and fen scrubs (F9.2), inland cliffs and rock pavements (H3.2), and temperate shrub heathlands (F4), although the latter is not officially classified under a EUNIS code. Certain habitats have not been attributed to EUNIS codes, such as schist quarries historically exploited for road construction, and apple orchards. Thus, in total, eight sites of calcareous grasslands (E1.2), eight sites of lowland hay meadows (E2.22), and eight sites of perennial tall-herb communities in humid habitats (E5.41 - though one of the sites was officially attributed to the category G1c1a - Plantations of *Populus* along river) were selected. Additionally, two orchards, one sandstone and one calcareous quarry, one heathland, one woodland, one permanent mesotrophic pasture and one site combining woodland, hay meadows and *Salix* and fen scrub were selected. To maintain consistency in our sampling protocol, and due to the insufficient presence of the latter habitats in the National Park to include eight sites per habitat type, these last eight sites were grouped under the category 'other habitats' for the purposes of presenting the results in figures. Although ecologically heterogeneous, the inclusion of these eight additional sites aims to provide a more comprehensive overview of bee diversity within the ESEMNP.

**Sampling protocol**

From April to August, each of the 32 study sites was visited once per month, yielding five sampling events per site over the course of the survey. This period corresponds to the peak activity of most wild bee species in Belgium (Duchenne et al. 2020). Sampling followed a uniform protocol to ensure comparability across sites. During each visit, bees were collected for a total of 40 minutes of effective sampling within the plot. This time exceeds the average foraging trip duration of most bee species (Gathmann and Tscharntke 2002). This time frame is thus sufficient to ensure that individuals regularly using the site had the opportunity to complete at least one trip between their nest and the sampling location during the observation period. The timer was halted whenever a specimen was captured to allow for its transfer into a vial and for the recording of collection details, after which active searching resumed.

Bees were sampled exclusively using a net. This method is particularly effective for Apidae and Megachilidae, which are generally caught more often with nets than with coloured pan traps; in contrast, Halictidae tend to be more frequently recorded in traps (Leclercq et al. 2022, Vandaudenard 2023). Netting also favours the capture of larger and slower-flying taxa, such as bumblebees (*Bombus* spp.) (Prendergast et al. 2020). Although combining netting with pan traps can broaden species detection, pan traps were deliberately excluded from this survey. In the ESEMNP, unattended traps placed between monthly visits are prone to theft or disturbance, either by people or by large herbivores. Moreover, prolonged immersion in water often damages smaller specimens, making morphological traits - especially those linked to hair colour or appearance - difficult to assess. The use of pan traps may also lead to considerable mortality of non-target insects, raising ethical concerns. An additional advantage of netting is that it allows direct observation and recording of the floral resources visited by captured individuals.

Sampling was conducted using a flexible transect method, in which the collector moved freely within the site, targeting areas with floral resources or potential nesting habitats rather than following a fixed straight line. This adaptive approach has been shown to increase the range of species detected, as it concentrates effort in zones with high bee activity (Westphal et al. 2008). Captured specimens were placed in vials containing paper moistened with ethyl acetate to ensure rapid euthanasia. For each individual collected, information was recorded on location (GPS coordinates, altitude, site code), behaviour (e.g., flying or interacting with a plant), and, when applicable, the plant species being visited. Sampling was restricted to favourable weather conditions, taking place between 9 AM and 5 PM, at temperatures above 15 °C, and in the absence of rainfall.

**Specimen curation and analyses**

The day following euthanasia, specimens were mounted by inserting a pin dorsally through the mesosoma. In males, genitalia were extracted from the metasoma using fine entomological pins, as these structures are frequently critical for reliable species-level identification (e.g. Rasmont et al. 2021, Wood 2023). Species determinations were made under a binocular microscope using standard taxonomic keys, and all specimens underwent verification by specialists for each bee family. Apidae identifications were reviewed by Frédéric Carion, Guillaume Ghisbain, and Achik Dorchin (identification keys: Smit 2018, Rasmont et al. 2021); Megachilidae by Clément Tourbez (identification key: Pauly 2019b); Halictidae by Thomas Brau and Simone Flaminio (identification keys: Amiet et al. 2001, Pauly 2019a); Andrenidae by William Fiordaliso, Thomas Wood and Maxence Gérard (identification key: Wood 2023); Colletidae by Romain Le Divelec (identification key: Amiet et al. 2014); and Melittidae by Maxence Gérard (identification key: Amiet et al. 2020).

Once the identification process was complete, we assessed sampling completeness by constructing a species accumulation curve with the iNEXT package (Hsieh et al. 2016). This approach enables visualisation of species detection rates relative to sampling effort and supports extrapolation of the additional effort required to encounter further species. The total species richness for the region was then estimated following Chao’s methodology (Chao 1984, Chao 1987).

## Results and Discussion

**Diversity and abundance of species**

**Figure 3.**
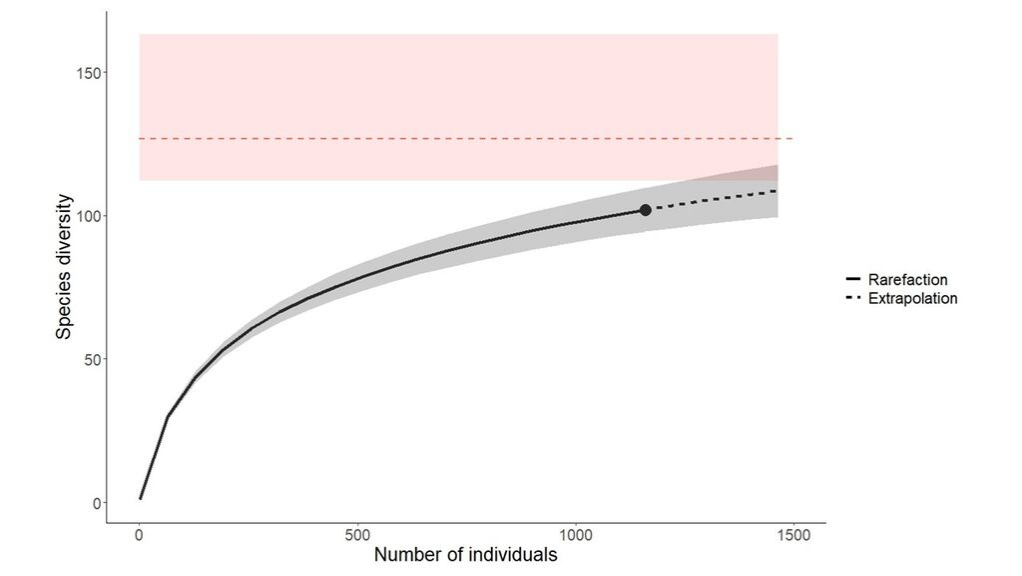
Species accumulation curve showing the estimated richness of specimens collected in the Entre-Sambre-et-Meuse National Park (Belgium). Sampling effort is expressed as the number of specimens collected on the x-axis. The dotted line indicates the expected number of species (y-axis) in relation to the number of specimens. Total species richness and its 95% confidence interval (in red) were estimated using the Chao method.

Our standardised sampling effort yielded 1,159 bee specimens, corresponding to 102 species out of the 419 known to occur in Belgium, representing 24.3% of the national fauna. For comparison, a study conducted with a similar protocol and over a comparable time frame in the Semois Valley National Park (Belgium) recorded 1,119 individuals belonging to 120 species, representing 28,6% of the national fauna (Gérard et al. 2025). Although the number of specimens collected is nearly identical, species richness in our survey was 15% lower than that documented in the Semois Valley. Furthermore, the species accumulation curve (Fig. 3) suggests that many taxa remain undetected in the ESEMNP. Using the richness estimator of Chao (1984), Chao (1987), we predict a total of 127 species for the National Park, with a confidence interval ranging from 112 to 163 species (Fig. 3). This indicates that the present dataset likely captures only 62.6–91% of the true fauna. The relatively wide standard error could further emphasise the need for additional sampling to obtain a more accurate estimate. However, compared to the study in the SVNP, the standard error is narrower, indicating that we should be closer to the actual species richness (Gérard et al. 2025). When compared to other protected areas of similar size in temperate Europe, surveyed during one spring and one summer, the species richness observed here appears slightly lower than in Wielkopolska National Park, Poland (n = 110; Banaszak-Cibicka et al. 2018), or in the Semois Valley National Park, Belgium (n = 120; Gérard et al. 2025). Nevertheless, it is noteworthy that, despite the pivotal role of bees in terrestrial ecosystems and the extensive network of protected areas across Europe, studies employing standardised protocols to assess their bee diversity and community composition remain particularly scarce (Chowdhury et al. 2023).

**Figure 4.**
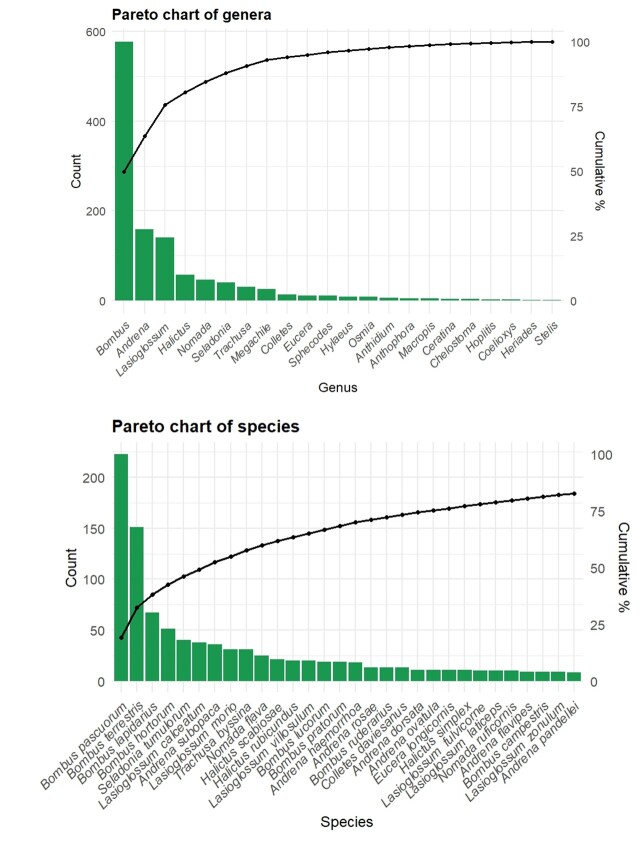
Frequency of the 22 genera (A) and 30 most abundant species (B) and their cumulative percentage contribution. Categories are ordered from most to least frequent along the x-axis.

In terms of community composition, bumblebees (genus *Bombus*, Apidae) dominated the assemblage, representing nearly half of all individuals (49.87%, n = 578; Fig. 4). The most frequently collected species was *Bombus pascuorum* (19.24%, n = 223; Fig. 4), followed by members of the subgenus Bombus sensu stricto (13.11%, n = 152; Fig. 4), a group in which females are hardly distinguishable without molecular or semiochemical tools (Rasmont et al. 2021). Historically, the dominance of *B. pascuorum* and *B. terrestris* (the most dominant member of *Bombus* sensu stricto in Belgium) was less evident, but their current prevalence could reflect the combined effects of rising temperatures and habitat degradation (Bommarco et al. 2011, Vray et al. 2019, Herbertsson et al. 2021). *Bombus lapidarius* ranked third among bumblebees (5.78%, n = 67; Fig. 4). The prominence of this small number of widespread taxa may reflect both the ecological flexibility and tolerance to changing environmental conditions of these particular species (Rasmont et al. 2015). Moreover, this strong dominance of two generalist bumblebee species - together representing approximately one third of the observed individuals and primarily interacting with widespread plant taxa such as *Taraxacum* spp. - suggests an ongoing homogenisation and simplification of plant–pollinator communities. Such patterns are increasingly reported across human-modified landscapes and reflect a shift towards communities dominated by disturbance-tolerant generalists at the expense of more specialised species (Deguines et al. 2016, Herbertsson et al. 2021). This biotic homogenisation constitutes a major threat to biodiversity, as it reduces functional diversity and ecological resilience, and has been identified as a key driver of biodiversity loss in the IPBES Global Assessment (IPBES 2019). However, part of this pattern could also arise from methodological constraints, since active netting tends to favour the capture of larger, more conspicuous social species over smaller solitary taxa (Prendergast et al. 2020). Among solitary bees, the most abundant species was *Seladonia tumulorum* (Halictidae), accounting for 3.45% of all collected bees (n = 40; Fig. 4). This polylectic species is widespread in Belgium, with stable populations (Pauly 2019a), which may explain its prevalence in the dataset. Although it typically occurs across a broad range of habitats, the majority of specimens in this study (62.5%) were collected in calcareous grasslands. The second most numerous solitary species was *Lasioglossum calceatum* (3.28%, n = 38; Fig. 4), which has an extended flight season in Belgium, from March to October. It commonly occurs in meadows; here, however, the 38 specimens were almost evenly distributed across the four habitat types. This species is often associated with *Taraxacum* flowers, which have actually been observed in each habitat type. The third rank in abundance among solitary bees is represented by *Andrena subopaca* (each 3.11%, n = 36; Fig. 4), a ubiquitous and polylectic species.

The structure of bee assemblages can also be assessed through the proportion of parasitic species. Brood parasitic bees, which exploit the nests of other species to reproduce, are present only where host populations are sufficiently abundant and supported by adequate floral resources. Because of their reliance on hosts, they occupy a higher trophic level within bee communities and are considered valuable indicators of ecological integrity (Michener 2007, Sheffield et al. 2013). Sheffield et al. (2013) suggested that when parasites represent more than 20% of the species recorded in a survey, this indicates an unusually high prevalence of the group. In our dataset, 22 of the 101 species (21.8%) were either brood parasites (*Coelioxys*, *Nomada*, *Sphecodes*, *Stelis*) or inquilines (bumblebees of the subgenus Psithyrus). This proportion is comparable to that reported for the SVNP and slightly exceeds the threshold proposed by Sheffield et al. (2013), indicating a notable representation of parasitic taxa in the study area. Such a pattern may reflect both a diverse and stable host community, especially considering that net sampling likely led to the underrepresentation of other parasitic species.

The sampling yielded 29 singletons, accounting for 28.4% of all species; a proportion very similar to that reported for the SVNP (28.3%), but generally higher than values typically observed in wild bee communities (e.g. Williams et al. 2001, Fiordaliso et al. 2022). The predominance of singletons is consistent with the shape of the species accumulation curve, which suggests that additional sampling would be necessary to achieve a more complete assessment of bee diversity in the ESEMNP. While some singletons may genuinely reflect naturally rare species with small populations, others are likely attributable to methodological limitations. The park hosts a mosaic of heterogeneous microhabitats, partially represented in the “Other habitats” category, but many occur as small, fragmented patches that may restrict species’ access to essential floral resources and thus reduce local abundance. Moreover, species with short flight periods are more difficult to detect when each site is sampled only once per month, further lowering the probability of encountering them, particularly for early-emerging species that fly before April in our protocol. Taken together, these factors indicate that the present survey likely underestimates the true bee diversity of the park.

**Threatened species and habitat specificities**

**Figure 5.**
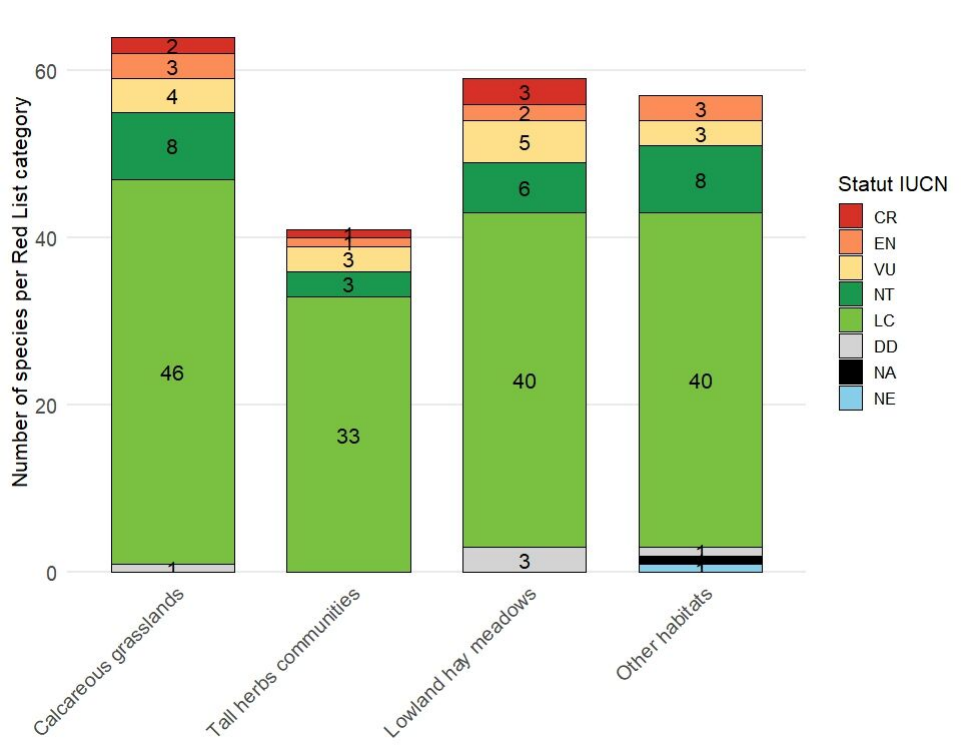
Distribution of species across the four habitat types, grouped by their Belgian Red List status (Drossart et al. 2019). CR: Critically Endangered, EN: Endangered, VU: Vulnerable, NT: Near Threatened, LC: Least Concern, DD: Data Deficient, NA: Not Applicable, NE: Not Evaluated.

Of the 102 bee species identified, 17 are classified as threatened at the national level according to the Belgian Red List (CR: Critically Endangered, *n* = 5; EN: Endangered, *n* = 4; VU: Vulnerable, *n* = 9; Drossart et al. 2019), representing 16.7% of the total assemblage (Fig. 5). An additional 12 species are listed as Near Threatened (NT). Among the most noteworthy taxa, *Bombus sylvarum*, *Halictus quadricinctus*, *Lasioglossum costulatum* and *Megachile argentata* are classified as Critically Endangered. The ecology of two of these species - absent from previous surveys in SVNP - is detailed below (Box 1). A full list of species collected and their Red List status in Belgium is provided in Table 1. We also recorded 9 species that are legally protected in Wallonia within the boundaries of the National Park. The most frequently encountered were *Trachusa byssina* (*n* = 31) and *Eucera longicornis* (*n* = 11). While scarce in the northern part of the country, *T. byssina* can be locally abundant in southern regions, especially in summer and in thermophilous habitats. Consistently, 60% of the individuals recorded here were collected in calcareous grasslands. *E. longicornis* is a spring to early-summer species strongly associated with Fabaceae-rich meadows, from which it collects the bulk of its pollen (Hennessy et al. 2020). Consistent with this ecology, 10 out of the 11 specimens were captured in lowland hay meadows.

In calcareous grasslands, 64 species were recorded among 295 individuals, 15 of which were exclusive to this habitat type in our survey. This habitat also supported a notable number of threatened species, with eight Red-listed taxa: two Critically Endangered (CR), three Endangered (EN), and three Vulnerable (VU). Three species were found exclusively in calcareous grasslands: *Megachile argentata* (*n* = 4, CR; Box 1), *Anthophora retusa* (*n* = 1, EN), and *Coelioxys mandibularis* (*n* = 1, VU). Beyond the first species already detailed in Box 1, *A. retusa* is relatively rare in Belgium and is undergoing declines in western Europe (Chorein 2007, Rasmont and Dehon 2015). In addition, calcareous grasslands hosted eight Near Threatened (NT) species. Altogether, 26.6% of the species recorded in this habitat are thus of conservation concern, and seven parasitic species were found exclusively here. Pauly and Vereecken 2018 investigated bee communities over four years in the calcareous grasslands of Han-sur-Lesse, a municipality located approximately 40 km east of the ESEMNP. Of the nine threatened species recorded in our study, only four were also observed in Han-sur-Lesse. Conversely, they reported seven threatened species that were absent from our survey. These findings underscore both the particularly high diversity of this habitat type and the marked differences that can occur even across relatively short spatial scales.

Similarly, lowland hay meadows supported a substantial number of species, with 59 species recorded among 331 specimens, 13 of which were found exclusively in this habitat. This environment harboured the highest number of threatened species, including three Critically Endangered, two Endangered, and five Vulnerable taxa. Four species were collected only in this habitat: *Halictus quadricinctus* (n = 1, CR), *Lasioglossum costulatum* (n = 1, CR), *Megachile alpicola* (n = 1, VU), and *Megachile leachella* (n = 1, VU). The occurrence of *M. leachella* is noteworthy, as this species is mainly confined to coastal sand dunes and is rarely observed inland (Praz and Bénon 2023). Interestingly, no sandy habitat was present at the collection site, which may suggest a broader habitat tolerance than previously assumed. Finally, with six Near Threatened species, lowland hay meadows also hold a particularly high proportion (27.1%) of species of conservation concern.

The ‘other habitats’ category harboured a substantial number of species (57 species out of 289 specimens), although with comparatively fewer threatened species (n = 7). Nine species were recorded exclusively in this category, including only one threatened species: *Bombus rupestris* (n = 1, EN). Because the ‘other habitats’ category is heterogeneous, the exact environments where species occurred varied. For example, heathlands can host specialised species, as they were the only habitat where we recorded *Andrena fuscipes*, a solitary summer-flying bee with an oligolectic diet restricted to Ericaceae (Exeler et al. 2010). In addition, two very rare species were recorded in this habitat category: *Andrena fulvata* (NA) and *Andrena rufula* (not evaluated on the Red List Drossart et al. 2019). The record of *Andrena fulvata* on *Rubus idaeus* in a heathland habitat in mid-May provides further evidence for the recent occurrence of this continental species in Belgium. *Andrena fulvata* is primarily distributed in central and eastern Europe and has only recently been documented in the country (Vertommen et al. 2024), potentially reflecting a recent expansion of its distribution range. This species is largely polylectic and is associated with a wide range of habitats, with a tendency towards open habitats within forested landscapes (Westrich 1989) – which corresponds to the habitat in which the individual was collected. *Andrena rufula* has been collected on *Prunus cerasifera* in an orchard in early April, which is consistent with the phenology and habitat preferences reported for this species. It is known from southern and central Europe and has recently been reported as new for Belgium, suggesting a northward expansion of its range (Wood 2023). Our record adds to the very limited number of confirmed observations and supports the view that the species occurs in thermophilous open habitats embedded within forested landscapes. The association with a flowering tree is in line with previous indications of a polylectic foraging behaviour with a preference for trees and shrubs, although floral preferences in this species remain poorly documented (Kocourek 1966).

The tall-herb communities of humid habitats harboured the lowest bee diversity, with only 41 species, two exclusive species, 244 specimens, and five threatened taxa. Their dense vegetation and moist soil likely provide few suitable nesting sites and is dominated by late-flowering plants, many of which are poor forage resources for bees, such as nettles (*Urtica dioica*). This combination reduces floral diversity and creates a limited temporal overlap with bee activity, making the habitat less attractive than more open, flower-rich meadows. In addition, the selection of study sites was based on existing Natura 2000 mapping and EUNIS habitat codes, some of which were established more than a decade ago. Site verification took place during winter, prior to the start of the vegetation period. As a result, certain sites were later found to be in poorer conservation condition during the growing season, leading to reduced floral diversity. This limitation may have influenced the representativeness of floral resources across sites. One of the threatened species were exclusive to this habitat: *Andrena helvola*. This early-flying, polylectic species has a marked preference for the pollen of shrubs and trees, often found around tall-herb communities (Wood and Roberts 2017). The other habitat-specific species is also of particular interest: *Macropis europaea*, which is legally protected in Belgium. This species specialises on *Lysimachia* spp., summer-flowering plants species typical of wet habitats. As with many oligolectic bees, *M. europaea* is not common but can occur locally in relatively high numbers (Drossart et al. 2019).

Finally, 18 species were recorded across all four habitat categories, suggesting a broad ecological niche. Most of these species are abundant in Belgium and listed as Least Concern on the Belgian Red List (Drossart et al. 2019), but the bumblebee *Bombus ruderarius* - classified as Endangered (EN) - was also present in all four habitats. This species has undergone a marked decline in western Europe over recent decades (Benton 2008, Nieto et al. 2014). In Belgium, it was already considered to be in strong decline at the end of the 20^th^ century (Rasmont et al. 1993). Although never common in the country, its relative abundance dropped from 2.34% of bumblebee records between 1910 and 1930 to just 0.19% between 1990 and 2016 (Rollin et al. 2020). The persistence of *B. ruderarius* in the ESEMNP therefore represents a key conservation concern.

## Box 1. Insights into two Critically Endangered species of the ESEMNP

### Halictus quadricinctus (Fabricius, 1776)

**Figure 6.**
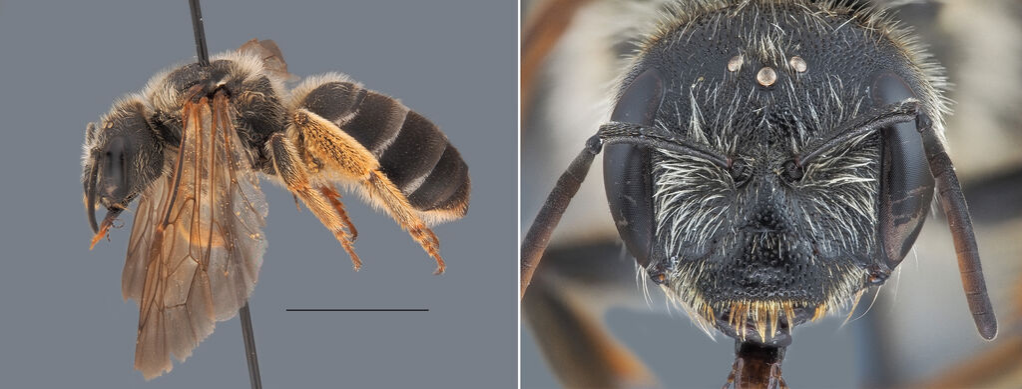
*Halictus quadricinctus*, ♀. Habitus in lateral view and head in frontal view. Scale bar: 5 mm. Photo credit: Paolo Rosa.

**Diagnosis**: This species is the largest *Halictus* in the country (females 14–16 mm, males 13–15 mm). In females, the scutum is large, with very sparse punctuation in the central area. In males, the metasoma gradually widens posteriorly, and the antennae bear long, characteristic ventral setae (Fig. 6).

**Ecology**: This is a trans-Palearctic species, occuring from Fennoscandia and the Iberian Peninsula through Central Asia, and extending as far east as northern and central China. During the last decades, this species was particularly rare in Belgium, and was only abundant in the Campine region. Recently, more occurrences are recorded throughout the country, in shrublands, grasslands, and thermophilous habitats like slag heaps, which are common in Hainaut’s Industrial backbone. The phenology of this species spans from early spring to late summer and is largely polylectic, collecting pollen from Asteraceae or Boraginaceae families.

**Threats and conservation**: Both Rasmont et al. (1993) and Pauly (2019a) reported that this species has undergone a marked decline in Belgium. Over the last century, its populations have been severely impacted by the combined effects of urbanization, habitat fragmentation, and grassland eutrophication resulting from agricultural intensification.

### Megachile argentata (Fabricius, 1793)

**Figure 7.**
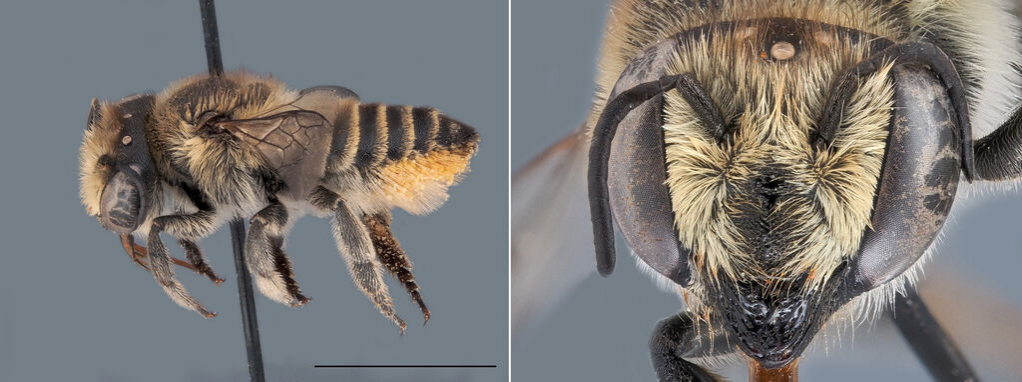
*Megachile argentata*, ♀. Habitus in lateral view and head in frontal view. Scale bar: 5 mm. Photo credit: Paolo Rosa.

**Diagnosis**: Among Belgian species, this bee is distinctive in being one of only two *Megachile* species whose females possess a white ventral scopa (Fig. 7). The other species, *M. leachella*, can be distinguished by the different punctation density on tergites 4 and 5, as well as by the clearly separated grey areas on tergite 6. Males are characterised by toothed anterior coxae - a rare trait among Belgian *Megachile* - and by sternite 4 bearing a median swelling on the apical margin, which separates them from *M. leachella* males.

**Ecology**: *Megachile argentata* occurs across Central Asia, Morocco, and Southern Europe, with its range expanding into central and Northern Europe (Praz and Bénon 2023). In Belgium, it was formerly considered rare, but records have increased noticeably in recent years. The species flies from May to August and is polylectic, showing more frequent interactions with plants of the Fabaceae, Lamiaceae and Asteraceae families (Westrich 1989, Praz 2017).

**Threats and conservation**: Although classified as Critically Endangered on the Belgian bee Red List (Drossart et al. 2019) due to its very limited distribution at the time, this species appears to have shown signs of population increase in recent years. In Belgium, it was initially confined to dry, warm habitats such as quarries and slag heaps, but it may now be benefiting from rising temperatures.

## Conclusion

Through the survey of wild bees in the Entre-Sambre-et-Meuse National Park, a total of 102 species were recorded. Among them, 17 species are considered threatened according to the Belgian Red List: 9 vulnerable (VU), 4 endangered (EN), and 4 critically endangered (CR). This species diversity along with the substantial proportion of threatened species highlights the critical need for the local conservation of the environments they were found in. Numerous studies have explored how conservation measures can mitigate the observed decline of wild bees (Drossart and Gérard 2020, Klaus et al. 2024), including in Belgium specifically (Schatz et al. 2021). Within this National Park, calcareous grasslands clearly play a central role in wild bee conservation. This thermophilous habitat provides early and abundant floral resources, making it critical to protect through practices such as short but intensive ovine grazing, reduced honey bee hive densities, and minimal use of agrochemicals in surrounding areas. Targeted management of sites hosting high numbers of threatened species is particularly important; for example, one lowland hay meadow site (“Centre géophysique”) harboured two critically endangered (CR) and two vulnerable (VU) species across less than 1.5 ha. Finally, several sites classified under the ‘other habitats’ category, such as heathlands - which are relatively uncommon within the National Park - warrant particular attention, as they supported several species not recorded elsewhere. Initiatives such as the establishment of this National Park can therefore both raise awareness and actively contribute to the protection of biodiversity, especially in a highly urbanized country like Belgium, where habitats are strongly fragmented.

## Figures and Tables

**Table 1.** Inventory of species collected in the Entre-Sambre-et-Meuse National Park (Belgium) in 2025. The table reports the proportion of individuals captured in each sampled habitat, together with the total number of specimens per species and their Belgian Red List status. CR: Critically Endangered, EN: Endangered, VU: Vulnerable, NT: Near Threatened, LC: Least Concern, DD: Data Deficient, NA: Not Applicable, NE: Not Evaluated.

**Taxon**	**IUCN Status**	**Protected in Wallonia**	**Calcareous grasslands**	**Tall-herb communities**	**Lowland hay meadows**	**Other habitats**	**Total**
** Andrenidae **							
*Andrena angustior*	NT	No	2	0	1	1	**4**
*Andrena chrysosceles*	LC	No	1	0	1	4	**6**
*Andrena cineraria*	LC	No	1	0	1	0	**2**
*Andrena dorsata*	LC	No	7	1	0	3	**11**
*Andrena falsifica*	DD	No	1	0	1	0	**2**
*Andrena flavipes*	LC	No	1	1	0	7	**9**
*Andrena fulva*	LC	No	0	1	0	1	**2**
*Andrena fulvago*	NT	No	0	0	1	0	**1**
*Andrena fulvata*	NA	No	0	0	0	1	**1**
*Andrena fuscipes*	LC	Yes	0	0	0	5	**5**
*Andrena gravida*	LC	No	4	0	1	1	**6**
*Andrena haemorrhoa*	LC	No	4	2	11	1	**18**
*Andrena helvola*	VU	No	0	2	0	0	**2**
*Andrena labialis*	NT	Yes	0	0	2	0	**2**
*Andrena labiata*	LC	No	0	0	1	0	**1**
*Andrena lathyri*	NT	No	3	0	0	0	**3**
*Andrena minutula*	LC	No	1	1	3	2	**7**
*Andrena minutuloides*	DD	No	0	0	1	0	**1**
*Andrena nigroaenea*	LC	No	1	0	0	0	**1**
*Andrena ovatula*	NT	No	7	0	0	4	**11**
*Andrena pandellei*	VU	No	5	0	3	0	**8**
*Andrena proxima*	LC	No	0	0	0	1	**1**
*Andrena rosae*	LC	No	0	11	1	1	**13**
*Andrena rufula*	NE	No	0	0	0	1	**1**
*Andrena subopaca*	LC	No	4	18	11	3	**36**
*Andrena ventralis*	LC	No	0	1	1	0	**2**
*Andrena viridescens*	LC	No	0	0	1	0	**1**
*Andrena wilkella*	NT	No	1	0	0	1	**2**
** Apidae **							
*Anthophora furcata*	LC	No	0	3	0	1	**4**
*Anthophora retusa*	EN	Yes	1	0	0	0	**1**
*Bombus bohemicus*	NT	No	0	0	0	2	**2**
*Bombus campestris*	VU	No	0	5	2	2	**9**
*Bombus hortorum*	NT	No	5	19	8	18	**50**
*Bombus hypnorum*	LC	No	1	2	2	2	**7**
*Bombus lapidarius*	LC	No	13	7	41	6	**67**
*Bombus lucorum*	NT	No	4	3	2	10	**19**
*Bombus pascuorum*	LC	No	31	59	67	66	**223**
*Bombus pratorum*	LC	No	3	4	1	11	**19**
*Bombus ruderarius*	EN	No	4	1	6	1	**12**
*Bombus rupestris*	EN	No	0	0	0	1	**1**
*Bombus* s. str. spp.	LC	No	28	37	40	47	**152**
*Bombus sylvarum*	CR	Yes	4	1	1	0	**6**
*Bombus sylvestris*	LC	No	0	3	0	5	**8**
*Bombus vestalis*	NT	No	0	1	0	1	**2**
*Ceratina cyanea*	LC	No	2	0	0	2	**4**
*Eucera longicornis*	VU	Yes	1	0	10	0	**11**
*Nomada conjugens*	LC	No	0	0	1	0	**1**
*Nomada fabriciana*	LC	No	1	0	0	2	**3**
*Nomada flava*	LC	No	3	0	20	2	**25**
*Nomada flavogutta*	LC	No	0	1	1	0	**2**
*Nomada marshamella*	LC	No	1	0	0	1	**2**
*Nomada ruficornis*	LC	No	2	4	3	1	**10**
*Nomada sheppardana*	LC	No	1	0	0	0	**1**
*Nomada zonata*	LC	No	1	0	0	1	**2**
** Colletidae **							
*Colletes daviesanus*	LC	No	1	9	3	0	**13**
*Hylaeus communis*	LC	No	1	2	1	1	**5**
*Hylaeus confusus*	LC	No	0	0	0	1	**1**
*Hylaeus gibbus*	DD	No	0	0	1	0	**1**
*Hylaeus gredleri*	DD	No	0	0	0	1	**1**
** Halictidae **							
*Halictus maculatus*	VU	No	0	1	0	3	**4**
*Halictus quadricinctus*	CR	No	0	0	1	0	**1**
*Halictus rubicundus*	LC	No	15	2	3	0	**20**
*Halictus scabiosae*	LC	No	4	9	8	0	**21**
*Halictus simplex*	EN	No	5	0	1	5	**11**
*Lasioglossum albipes*	NT	No	2	0	4	2	**8**
*Lasioglossum calceatum*	LC	No	12	11	7	8	**38**
*Lasioglossum costulatum*	CR	No	0	0	1	0	**1**
*Lasioglossum fulvicorne*	LC	No	3	1	2	4	**10**
*Lasioglossum laticeps*	LC	No	3	1	6	0	**10**
*Lasioglossum lativentre*	LC	No	0	2	1	2	**5**
*Lasioglossum leucozonium*	LC	No	2	0	3	2	**7**
*Lasioglossum morio*	LC	No	23	3	1	4	**31**
*Lasioglossum nitidulum*	LC	No	1	0	0	0	**1**
*Lasioglossum pauxillum*	LC	No	0	0	1	0	**1**
*Lasioglossum villosulum*	LC	No	1	0	10	9	**20**
*Lasioglossum zonulum*	LC	No	0	4	4	1	**9**
*Seladonia tumulorum*	LC	No	26	1	5	8	**40**
*Sphecodes ephippius*	LC	No	0	0	1	4	**5**
*Sphecodes ferruginatus*	LC	No	1	0	0	0	**1**
*Sphecodes gibbus*	LC	No	1	0	0	0	**1**
*Sphecodes hyalinatus*	LC	No	1	0	0	0	**1**
*Sphecodes monilicornis*	LC	No	0	1	0	1	**2**
*Sphecodes reticulatus*	LC	No	1	0	0	0	**1**
** Megachilidae **							
*Anthidium manicatum*	LC	No	2	0	0	0	**2**
*Anthidium oblongatum*	LC	No	2	0	0	0	**2**
*Anthidium punctatum*	LC	Yes	1	0	0	1	**2**
*Chelostoma florisomne*	LC	No	0	0	0	4	**4**
*Coelioxys mandibularis*	VU	Yes	1	0	0	0	**1**
*Coelioxys rufescens*	NT	Yes	1	0	0	0	**1**
*Heriades truncorum*	LC	No	1	0	0	0	**1**
*Hoplitis claviventris*	VU	No	2	0	0	1	**3**
*Megachile alpicola*	VU	No	0	0	1	0	**1**
*Megachile argentata*	CR	No	4	0	0	0	**4**
*Megachile centuncularis*	LC	No	0	2	1	0	**3**
*Megachile leachella*	VU	No	0	0	1	0	**1**
*Megachile ligniseca*	LC	No	1	1	0	0	**2**
*Megachile versicolor*	LC	No	5	0	3	0	**8**
*Megachile willughbiella*	LC	No	4	0	3	0	**7**
*Osmia leaiana*	LC	No	1	1	2	4	**8**
*Stelis punctulatissima*	LC	No	0	0	1	0	**1**
*Trachusa byssina*	LC	Yes	18	0	9	4	**31**
** Melittidae **							
*Macropis europaea*	LC	Yes	0	5	0	0	**5**

## References

[B13532275] Adriaens Dries, Honnay Olivier, Hermy Martin (2006). No evidence of a plant extinction debt in highly fragmented calcareous grasslands in Belgium. Biological Conservation.

[B13749160] Amiet F., Hermann M., Müller A., Neumeyer R. (2001). Apidae 3: *Halictus*, *Lasioglossum*. Fauna Helvetica.

[B13749134] Amiet F., Müller A., Neumeyer R. (2014). Apidae 2: *Colletes*, *Dufourea*, *Hylaeus*, *Nomia*, *Nomioides*, *Rhophitoides*, *Rophites*, *Sphecodes*, *Systropha*. Fauna Helvetica.

[B13749152] Amiet F., Hermann M., Müller A., Neumeyer R. (2020). Apidae 5: *Ammobates*, *Ammobatoides*, *Anthophora*, *Biastes*, *Ceratina*, *Dasypoda*, *Epeoloides*, *Epeolus*, *Eucera*, *Macropis*, *Melecta*, *Melitta*, *Nomada*, *Pasites*, *Tetralonia*, *Thyreus*, *Xylocopa*. Fauna Helvetica.

[B13532967] Banaszak-Cibicka Weronika, Twerd Lucyna, Fliszkiewicz Monika, Giejdasz Karol, Langowska Aleksandra (2018). City parks vs. natural areas - is it possible to preserve a natural level of bee richness and abundance in a city park?. Urban Ecosystems.

[B13533064] Benton T. (2008). *Bombus ruderarius* (Müller, 1776): Current knowledge of its autecology and reasons for decline..

[B13532344] Biesmeijer J. C., Roberts S. P. M., Reemer M., Ohlemüller R., Edwards M., Peeters T., Schaffers A. P., Potts S. G., Kleukers R., Thomas C. D., Settele J., Kunin W. E. (2006). Parallel declines in pollinators and insect-pollinated plants in Britain and the Netherlands. Science.

[B13532988] Bommarco Riccardo, Lundin Ola, Smith Henrik G., Rundlöf Maj (2011). Drastic historic shifts in bumble-bee community composition in Sweden. Proceedings of the Royal Society B: Biological Sciences.

[B13532956] Chao A. (1984). Nonparametric estimation of the number of classes in a population.

[B13532947] Chao Anne (1987). Estimating the population size for capture-recapture data with unequal catchability. Biometrics.

[B13606853] Chorein A. (2007). Systématique et Chorologie des Anthophorini (Hymenoptera: Apidae) de Belgique et du Nord de la France, avec une première analyse de leurs sécrétions volatiles..

[B13532977] Chowdhury Shawan, Jennions Michael D., Zalucki Myron P., Maron Martine, Watson James E. M., Fuller Richard A. (2023). Protected areas and the future of insect conservation. Trends in Ecology & Evolution.

[B13748964] Deguines Nicolas, Julliard Romain, de Flores Mathieu, Fontaine Colin (2016). Functional homogenization of flower visitor communities with urbanization. Ecology and Evolution.

[B13532693] Diaz S., Settele J., Brondizio E. S. (2019). Summary for policymakers of the global assessment report on biodiversity and ecosystem services of the Intergovernmental Science-Policy Platform on Biodiversity and Ecosystem Services (IPBES)..

[B13532488] Dicks Lynn V., Breeze Tom D., Ngo Hien T., Senapathi Deepa, An Jiandong, Aizen Marcelo A., Basu Parthiba, Buchori Damayanti, Galetto Leonardo, Garibaldi Lucas A., Gemmill-Herren Barbara, Howlett Brad G., Imperatriz-Fonseca Vera L., Johnson Steven D., Kovács-Hostyánszki Anikó, Kwon Yong Jung, Lattorff H. Michael G., Lungharwo Thingreipi, Seymour Colleen L., Vanbergen Adam J., Potts Simon G. (2021). A global-scale expert assessment of drivers and risks associated with pollinator decline. Nature Ecology & Evolution.

[B13606821] Drossart M., Rasmont P., Vanormelingen P. (2019). Belgian Red List of Bees.

[B13532761] Drossart Maxime, Gérard Maxence (2020). Beyond the decline of wild bees: Optimizing conservation measures and bringing together the actors. Insects.

[B13532739] Duchenne François, Thébault Elisa, Michez Denis, Gérard Maxence, Devaux Céline, Rasmont Pierre, Vereecken Nicolas J., Fontaine Colin (2020). Long‐term effects of global change on occupancy and flight period of wild bees in Belgium. Global Change Biology.

[B13532752] Exeler Nina, Kratochwil Anselm, Hochkirch Axel (2010). Does recent habitat fragmentation affect the population genetics of a heathland specialist, *Andrena
fuscipes* (Hymenoptera: Andrenidae)?. Conservation Genetics.

[B13607969] Fiordaliso W., Saiz S. R., Wood T. J. (2022). Inventaire et conservation des abeilles sauvages (Hymenoptera: Anthophila) du sillon industriel hainuyer (Belgique). Belgian Journal of Entomology.

[B13532730] Gathmann Achim, Tscharntke Teja (2002). Foraging ranges of solitary bees. Journal of Animal Ecology.

[B13532456] Gekière Antoine, Gérard Maxence, Potts Simon G, Michez Denis, Ghisbain Guillaume (2025). Underlying mechanisms shaping wild bee decline. Biological Journal of the Linnean Society.

[B13532466] Gérard Maxence, Vanderplanck Maryse, Wood Thomas, Michez Denis (2020). Global warming and plant–pollinator mismatches. Emerging Topics in Life Sciences.

[B13532475] Gérard Maxence, Fiordaliso William, Ferrais Louise, Fournier Chloé, Hairault Malo, Lheureux Lise, Rosa Paolo, Ghisbain Guillaume (2025). Wild bee diversity of the National Park of the Semois Valley (Belgium). Biodiversity Data Journal.

[B13532432] Geslin B., Gauzens B., Baude M., Dajoz I., Fontaine C., Henry M., Ropars L., Rollin O., Thébault E., Vereecken N. J. (2017). Massively introduced managed species and their consequences for plant–pollinator interactions. Advances in Ecological Research.

[B13532394] Ghisbain Guillaume, Thiery Wim, Massonnet François, Erazo Diana, Rasmont Pierre, Michez Denis, Dellicour Simon (2023). Projected decline in European bumblebee populations in the twenty-first century. Nature.

[B13532369] Ghisbain GUILLAUME (2023). The new annotated checklist of the wild bees of Europe (Hymenoptera: *Anthophila*). Zootaxa.

[B13532406] Ghisbain Guillaume, Chittka Lars, Michez Denis (2025). Bumblebees. Current Biology.

[B13532447] Goulson Dave, Nicholls Elizabeth, Botías Cristina, Rotheray Ellen L. (2015). Bee declines driven by combined stress from parasites, pesticides, and lack of flowers. Science.

[B13533055] Hennessy Georgia, Goulson Dave, Ratnieks Francis L. W. (2020). Population assessment and foraging ecology of nest aggregations of the rare solitary bee, *Eucera
longicornis* at Gatwick Airport, and implications for their management. Journal of Insect Conservation.

[B13532997] Herbertsson Lina, Khalaf Reem, Johnson Karin, Bygebjerg Rune, Blomqvist Sofia, Persson Anna S. (2021). Long-term data shows increasing dominance of *Bombus
terrestris* with climate warming. Basic and Applied Ecology.

[B13532938] Hsieh T. C., Ma K. H., Chao Anne (2016). iNEXT: an R package for rarefaction and extrapolation of species diversity (Hill numbers). Methods in Ecology and Evolution.

[B13748986] IPBES (2019). Global assessment report on biodiversity and ecosystem services of the Intergovernmental Science-Policy Platform on Biodiversity and Ecosystem Services.

[B13532335] Jacquemin Floriane, Violle Cyrille, Rasmont Pierre, Dufrêne Marc (2017). Mapping the dependency of crops on pollinators in Belgium. One Ecosystem.

[B13532814] Klaus Felix, Ayasse Manfred, Classen Alice, Dauber Jens, Diekötter Tim, Everaars Jeroen, Fornoff Felix, Greil Henri, Hendriksma Harmen P., Jütte Tobias, Klein Alexandra Maria, Krahner André, Leonhardt Sara D., Lüken Dorothee J., Paxton Robert J., Schmid-Egger Christian, Steffan-Dewenter Ingolf, Thiele Jan, Tscharntke Teja, Erler Silvio, Pistorius Jens (2024). Improving wild bee monitoring, sampling methods, and conservation. Basic and Applied Ecology.

[B13749679] Kocourek M. (1966). Prodromus der Hymenopteren der Tschechoslowakei. Pars 9: Apoidea, 1. Gattung Andrena. Acta Faunistica Entomologica Musei Nationalis Pragae Supplementum.

[B13532778] Leclercq N., Marshall L., Weekers T., Anselmo A., Benda D., Bevk D., Bogusch P., Cejas D., Drepper B., Galloni M., Gérard M., Ghisbain G., Hutchinson L., Martinet B., Michez D., Molenberg J. -M., Nikolic P., Roberts S., Smagghe G., Straka J., Vandamme P., Wood T. J., Vereecken N. J. (2022). A comparative analysis of crop pollinator survey methods along a large-scale climatic gradient. Agriculture, Ecosystems & Environment.

[B13532806] Michener C. (2007). Bees of the world.

[B13532361] Nieto A., Roberts S. P.M., Kemp J. (2014). European red list of bees.

[B13532303] Ollerton Jeff, Winfree Rachael, Tarrant Sam (2011). How many flowering plants are pollinated by animals?. Oikos.

[B13533080] Pauly A., Vereecken N. (2018). The wild bees of calcareous grasslands of Han-sur-Lesse (Hymenoptera: Apoidea)..

[B13532867] Pauly A. (2019). Abeilles de Belgique et des régions limitrophes (Insecta: Hymenoptera: Apoidea). Famille Halictidae.

[B13749101] Pauly A. (2019). Clés illustrées pour l'identification des abeilles de Belgique et des régions limitrophes (Hymenoptera: Apoidea) II. Megachilidae. https://geonature.arb-idf.fr/sites/default/files/articles/documents/ClefIdent/Hymenopteres/Pauly_2015_cl%C3%A9_Megachilidae_Belgique.pdf.

[B13532324] Potts Simon G., Biesmeijer Jacobus C., Kremen Claire, Neumann Peter, Schweiger Oliver, Kunin William E. (2010). Global pollinator declines: trends, impacts and drivers. Trends in Ecology & Evolution.

[B13532849] Praz Christophe J. (2017). Subgeneric classification and biology of the leafcutter and dauber bees (genus *Megachile* Latreille) of the Western Palearctic (Hymenoptera, Apoidea, Megachilidae). Journal of Hymenoptera Research.

[B13532858] Praz Christophe J., Bénon Dimitri (2023). Revision of the leachella group of Megachile
subgenus
Eutricharaea in the Western Palaearctic (Hymenoptera, Apoidea, Megachilidae): A renewed plea for DNA barcoding type material. Journal of Hymenoptera Research.

[B13532840] Prendergast Kit S., Menz Myles H. M., Dixon Kingsley W., Bateman Philip W. (2020). The relative performance of sampling methods for native bees: an empirical test and review of the literature. Ecosphere.

[B13606829] Rasmont P., Leclercq J., Jacob-Remacle A., Bruneau E. (1993). Bees for pollination..

[B13532626] Rasmont P., Pauly A., Terzo M. (2005). The survey of wild bees (Hymenoptera, Apoidea) in Belgium and France.

[B13532642] Rasmont P., Dehon M. (2015). *Anthophora* spp, *Melecta* spp, *Thyreus* spp.

[B13533008] Rasmont Pierre, Franzen Markus, Lecocq Thomas, Harpke Alexander, Roberts Stuart, Biesmeijer Koos, Castro Leopoldo, Cederberg Bjorn, Dvorak Libor, Fitzpatrick Una, Gonseth Yves, Haubruge Eric, Mahe Gilles, Manino Aulo, Michez Denis, Neumayer Johann, Odegaard Frode, Paukkunen Juho, Pawlikowski Tadeusz, Potts Simon, Reemer Menno, Settele Josef, Straka Jakub, Schweiger Oliver (2015). Climatic risk and distribution atlas of European bumblebees. BioRisk.

[B13532415] Rasmont P., Terzo M., Ghisbain G. (2021). Bumblebees of Europe and neighbouring regions.

[B13532514] Reverté Sara, Miličić Marija, Ačanski Jelena, Andrić Andrijana, Aracil Andrea, Aubert Matthieu, Balzan Mario Victor, Bartomeus Ignasi, Bogusch Petr, Bosch Jordi, Budrys Eduardas, Cantú‐Salazar Lisette, Castro Sílvia, Cornalba Maurizio, Demeter Imre, Devalez Jelle, Dorchin Achik, Dufrêne Eric, Đorđević Aleksandra, Fisler Lisa, Fitzpatrick Úna, Flaminio Simone, Földesi Rita, Gaspar Hugo, Genoud David, Geslin Benoît, Ghisbain Guillaume, Gilbert Francis, Gogala Andrej, Grković Ana, Heimburg Helge, Herrera‐Mesías Fernanda, Jacobs Maarten, Janković Milosavljević Marina, Janssen Kobe, Jensen Jens‐Kjeld, Ješovnik Ana, Józan Zsolt, Karlis Giorgos, Kasparek Max, Kovács‐Hostyánszki Anikó, Kuhlmann Michael, Le Divelec Romain, Leclercq Nicolas, Likov Laura, Litman Jessica, Ljubomirov Toshko, Madsen Henning Bang, Marshall Leon, Mazánek Libor, Milić Dubravka, Mignot Maud, Mudri‐Stojnić Sonja, Müller Andreas, Nedeljković Zorica, Nikolić Petar, Ødegaard Frode, Patiny Sebastien, Paukkunen Juho, Pennards Gerard, Pérez‐Bañón Celeste, Perrard Adrien, Petanidou Theodora, Pettersson Lars B., Popov Grigory, Popov Snežana, Praz Christophe, Prokhorov Alex, Quaranta Marino, Radchenko Vladimir G., Radenković Snežana, Rasmont Pierre, Rasmussen Claus, Reemer Menno, Ricarte Antonio, Risch Stephan, Roberts Stuart P. M., Rojo Santos, Ropars Lise, Rosa Paolo, Ruiz Carlos, Sentil Ahlam, Shparyk Viktor, Smit Jan, Sommaggio Daniele, Soon Villu, Ssymank Axel, Ståhls Gunilla, Stavrinides Menelaos, Straka Jakub, Tarlap Peeter, Terzo Michael, Tomozii Bogdan, Tot Tamara, van der Ent Leendert‐Jan, van Steenis Jeroen, van Steenis Wouter, Varnava Androulla I., Vereecken Nicolas J., Veselić Sanja, Vesnić Adi, Weigand Alexander, Wisniowski Bogdan, Wood Thomas J., Zimmermann Dominique, Michez Denis, Vujić Ante (2023). National records of 3000 European bee and hoverfly species: A contribution to pollinator conservation. Insect Conservation and Diversity.

[B13532658] Rollin Orianne, Vray Sarah, Dendoncker Nicolas, Michez Denis, Dufrêne Marc, Rasmont Pierre (2020). Drastic shifts in the Belgian bumblebee community over the last century. Biodiversity and Conservation.

[B13532669] Schatz Bertrand, Maxime Drossart, Mickael Henry, Benoît Geslin, Fabrice Allier, Colette Savajol, Maxence Gérard, Denis Michez (2021). Pollinator conservation in the context of global changes with a focus on France and Belgium. Acta Oecologica.

[B13533037] Sheffield Cory S., Pindar Alana, Packer Laurence, Kevan Peter G. (2013). The potential of cleptoparasitic bees as indicator taxa for assessing bee communities. Apidologie.

[B13749092] Smit J. (2018). Identification Key to the European Species of the Bee Genus Nomada Scopoli, 1770 (Hymenoptera: Apidae), Including 23 New Species. *Entomofauna Monographs*.

[B13532312] Tong Ze-Yu, Wu Ling-Yun, Feng Hui-Hui, Zhang Meng, Armbruster W Scott, Renner Susanne S, Huang Shuang-Quan (2023). New calculations indicate that 90% of flowering plant species are animal-pollinated. National Science Review.

[B13532717] Turley Nash E, Kania Sarah E, Petitta Isabella R, Otruba Elizabeth A, Biddinger David J, Butzler Thomas M, Sesler Valerie V, López-Uribe Margarita M (2024). Bee monitoring by community scientists: comparing a collections-based program with iNaturalist. Annals of the Entomological Society of America.

[B13532709] Vandaudenard T. (2023). Étude des populations d’abeilles sauvages du Parc naturel Viroin-Hermeton. http://hdl.handle.net/2078.1/thesis:42287.

[B13607958] Vertommen Win, Vanormelingen Pieter, D'Haeseleer Jens (2024). New and confirmed wild bee species (Hymenoptera: Apoidea: Apiformes) for the fauna of Belgium, with notes on the rediscovery of regionally extinct species. Belgian Journal of Entomology.

[B13532682] Vray Sarah, Rollin Orianne, Rasmont Pierre, Dufrêne Marc, Michez Denis, Dendoncker Nicolas (2019). A century of local changes in bumblebee communities and landscape composition in Belgium. Journal of Insect Conservation.

[B13532294] WallisDeVries Michiel F, Poschlod Peter, Willems Jo H (2002). Challenges for the conservation of calcareous grasslands in northwestern Europe: integrating the requirements of flora and fauna. Biological Conservation.

[B13532875] Westphal Catrin, Bommarco Riccardo, Carré Gabriel, Lamborn Ellen, Morison Nicolas, Petanidou Theodora, Potts Simon G., Roberts Stuart P. M., Szentgyörgyi Hajnalka, Tscheulin Thomas, Vaissière Bernard E., Woyciechowski Michal, Biesmeijer Jacobus C., Kunin William E., Settele Josef, Steffan-Dewenter Ingolf (2008). Measuring bee diversity in different European habitats and biogeographical regions. Ecological Monographs.

[B13534474] Westrich P. (1989). Die Wildbienen Baden-Württembergs.

[B13533046] Williams Neal M., Minckley Robert L., Silveira Fernando A. (2001). Variation in native bee faunas and its implications for detecting community changes. Conservation Ecology.

[B13532896] Wood Thomas J., Roberts Stuart P. M. (2017). An assessment of historical and contemporary diet breadth in polylectic *Andrena* bee species. Biological Conservation.

[B13606842] Wood TJ. (2023). The genus *Andrena* in Belgium: revisions, clarifications, and a key for their identification (Hymenoptera: Andrenidae). Belgian Journal of Entomology.

[B13532423] Zattara Eduardo E., Aizen Marcelo A. (2021). Worldwide occurrence records suggest a global decline in bee species richness. One Earth.

